# Systematic, network-based characterization of therapeutic target inhibitors

**DOI:** 10.1371/journal.pcbi.1005599

**Published:** 2017-10-12

**Authors:** Yao Shen, Mariano J. Alvarez, Brygida Bisikirska, Alexander Lachmann, Ronald Realubit, Sergey Pampou, Jorida Coku, Charles Karan, Andrea Califano

**Affiliations:** 1 Department of Systems Biology, Columbia University, New York, New York, United States of America; 2 DarwinHealth Inc, New York, New York, United States of America; 3 JP Sulzberger Columbia Genome Center, Columbia University, New York, New York, United States of America; 4 Department of Biomedical Informatics, Columbia University, New York, New York, United States of America; 5 Department of Biochemistry & Molecular Biophysics, Columbia University, New York, New York, United States of America; 6 Institute for Cancer Genetics and Herbert Irving Comprehensive Cancer Center, Columbia University, New York, New York, United States of America; 7 Motor Neuron Center and Columbia Initiative in Stem Cells, Columbia University, New York, New York, United States of America; Danish Cancer Society Research Center, DENMARK

## Abstract

A large fraction of the proteins that are being identified as key tumor dependencies represent poor pharmacological targets or lack clinically-relevant small-molecule inhibitors. Availability of fully generalizable approaches for the systematic and efficient prioritization of tumor-context specific protein activity inhibitors would thus have significant translational value. Unfortunately, inhibitor effects on protein activity cannot be directly measured in systematic and proteome-wide fashion by conventional biochemical assays. We introduce OncoLead, a novel network based approach for the systematic prioritization of candidate inhibitors for arbitrary targets of therapeutic interest. *In vitro* and *in vivo* validation confirmed that OncoLead analysis can recapitulate known inhibitors as well as prioritize novel, context-specific inhibitors of difficult targets, such as *MYC* and *STAT3*. We used OncoLead to generate the first unbiased drug/regulator interaction map, representing compounds modulating the activity of cancer-relevant transcription factors, with potential in precision medicine.

## Author summary

Most transcription factors are considered “undruggable” in conventional drug discovery. However, a large number of them are discovered to be key tumor dependencies. Thus, targeting these difficult targets has been a challenge for cancer drug discovery. Here, we introduce a novel method, OncoLead, that applies biological networks to identify candidate inhibitors that either directly or in-directly block the activities of these targets. This approach is confirmed by known target-inhibitor interactions in public databases. Furthermore, we predicted new inhibitors for *MYC* and *STAT3*, which are validated by *in vitro* assays.

## Introduction

While the number of high-value, candidate therapeutic target proteins has increased dramatically over the past five years, most of them lack a corresponding FDA-approved or late-stage investigational (i.e., clinically relevant) small-molecule inhibitor. Furthermore, a large number of these are considered undruggable and may thus benefit from small molecules inducing potent, albeit indirect inhibition, within a specific tumor context. For instance, ibrutinib, a Bruton’s Tyrosine Kinase (*BTK*) inhibitor, can effectively abrogate aberrant *NF-kB* activity in human B cells, with clinically relevant application to treatment of the ABC subtype of diffuse large B cell lymphoma [1].

A key problem in addressing this challenge is the lack of generalizable methodologies for the efficient and systematic prioritization of small molecule compounds as direct or indirect inhibitors of an arbitrary protein of interest. Throughout this manuscript, we will use the word ‘compound’ for short to refer to small molecule compounds. Consistently, by compound targets and compound activity we refer to the proteins targeted by the small molecule compound and its pharmacological activity, respectively. Indeed, high-throughput screens (HTS) mostly rely on *ad hoc*, experimental gene reporter assays, whose design, testing, optimization, and miniaturization is laborious and inefficient. In addition, most of these assays are limited to reporting on the activity of a single target protein or of a specific protein class (e.g., protein kinases [2]). Computational HTS approaches, such as quantitative structure activity relation (QSAR) analysis [3] and virtual screening [4], rely on availability of structural models for both the ligands and the target protein and thus on prior knowledge from related compound’s binding assays or from X-ray/NMR target structure elucidation [3]. For instance, the similarity ensemble approach (SEA), which predicts new target-ligand relationships based on their similarity to established target-ligand sets, is widely adopted [5]. However, results completely depend on the availability of ligand analogs, whose structure has been previously elucidated.

Critically, these methods lack cell-context specificity and are limited to assessing only direct, high-affinity binding compounds, thus missing small-molecule compounds that may indirectly modulate the activity of a target protein, as is the case for ibrutinib. These compounds cannot be assessed by QSAR, because they do not represent high-affinity ligands of the target protein of interest but rather of one of its major context-specific up-stream regulators. In addition, these methods are not effective for protein families that lack specific binding pockets, such as transcription factors (TFs) [6], even though these comprise many of the best established tumor dependencies. Indeed, TFs such as *ESR1*, *NOTCH1*, *MYC*, *GATA3*, and *ERG*, among many others, are frequently aberrantly activated in cancer [7]. In addition, many TFs have been recently elucidated as Master Regulators of tumor cell state, which are organized in highly interconnected modules or tumor checkpoints [8], including key synthetic lethal combinations, such as *STAT3*, *CEBPB*, and *CEBPD* in mesenchymal glioblastoma [9] or *CENPF* and *FOXM1* in malignant prostate carcinoma [10].

Recently, several perturbational strategies have been proposed to measure differential gene expression following systematic chemical perturbations of specific cell lines, such as the connectivity map (CMAP) [11] and the Library of Integrated Network-based Cellular Signatures (LINCS) [12]. However, since most small molecule compounds affect the activity rather than the expression of target proteins, these data cannot elucidate targets but rather their ability to modulate the entire gene expression signature of a cell. We recently introduced DeMAND, a method for the interrogation of cell context specific networks, to infer drug mechanism of action (MoA) [13]. While being very efficient to capture direct as well as indirect context-specific targets [13], DeMAND requires at least six gene expression profiles per compound. As a result, while it is very effective for elucidating the MoA of individual compounds of interest, it is not optimally suited to the reverse problem, i.e., prioritizing candidate protein inhibitors from large-scale perturbational profiles, especially when fewer than six perturbational profiles per compound are available.

We thus developed *OncoLead*, a novel and highly generalizable methodology for the efficient and systematic identification of small molecules that directly or indirectly inhibit a target protein of interest. *OncoLead* leverages the Virtual Inference of Protein activity by Enriched Regulon analysis (VIPER) algorithm [10, 14]—a network-based algorithm for the assessment of protein activity from gene expression data—to assess the effect of a panel of drugs on protein activity from individual expression profiles. We limit our analysis to ~7,000 regulatory proteins (RPs), including ~2,000 transcription factors (TFs) and ~5,000 signaling proteins (SIGs), whose regulatory ‘activity’ may be modulated by a small-molecule compound. While these represent only ~30% of the human genome, they capture an important component of relevant tumor dependencies that may benefit from targeted inhibitor availability.

Briefly, given two cellular states (e.g., baseline and compound-perturbed), OncoLead uses the differential expression of a protein’s transcriptional targets (*i*.*e*., its *regulon*) as an accurate and highly reproducible multiplexed endogenous reporter assay for its activity [15, 16]. For a given RP, the regulon comprises its context-specific direct or indirect transcriptional targets [17]. This approach is especially well suited to the screening of large libraries of compounds for two reasons: first, it can accurately infer compound-mediated protein activity modulation from a single perturbational profile (e.g., RNASeq following perturbation); second, its performance is essentially unaffected when RNASeq depth is reduced from 30M to 0.5M reads [18], thus allowing highly-multiplexed characterization of the activity of compounds at low cost.

We first show that OncoLead can effectively assess differential activity for established targets of the compound, even when these are not differentially expressed following compound’s perturbation. To accomplish this goal, we leveraged two public databases including the Connectivity MAP (CMAP) [11] and the Library of Integrated Network-based Cellular Signatures (LINCS) (http://lincs.hms.harvard.edu/), as well as one *in vivo* dataset, containing gene expression profiles (GEPs) obtained post-treatment from patients’ tumor tissue. For each cell line or tissue represented in the datasets, the analysis was performed using networks representing the transcriptional targets of the candidate compound-targeted proteins in tissue lineage-matched contexts. We used the algorithm to assemble the first comprehensive, cell-context-specific map of inhibitors targeting RPs. The associated resource, which includes a comprehensive map of RP-compound’s interactions, is available as a supplementary file linked to this publication. We then show that the algorithm is effective in elucidating novel tumor-specific inhibitors of undruggable targets. Specifically, OncoLead was highly effective in inferring novel breast-cancer-specific inhibitors of *MYC* and *STAT3*, which were experimentally validated.

## Results

### Context-specific mechanism of action for small molecule compounds can be described by network-based protein activity inferences

OncoLead assesses whether a compound is an effective inhibitor/activator of a given regulatory protein, based on its effect on the transcriptional level of the protein’s regulon—i.e. its set of direct and indirect transcriptional targets—to infer the regulatory protein’s differential activity; see Methods and [18]. For simplicity, we call compound’s mode-of-action (CMoA) to the full repertoire of proteins, whose activity is significantly affected following perturbation with the compound. These include both direct targets as well as context-specific downstream effectors of compound’s activity, and thus effectively representing the context-specific compound’s MoA.

Clearly, the accuracy of our inferences of protein activity depends on the quality of the protein regulons. Due to lineage specific chromatin remodeling and co-factor availability, protein regulons are highly cell context specific [19, 20]. In this work, we used the ARACNE algorithm [21] for context-specific inference of the regulatory network. We have previously shown that regulons inferred by ARACNE are particularly suited for VIPER analysis [18]. As shown in Table 1, ARACNE-based regulon inference was performed using tumor-context matched gene expression profiles (GEP) from The Cancer Genome Atlas consortium (TCGA) [22], and relevant tumor context matched GEO datasets [23], when available.

**Table 1:** Context-specific interactomes and the datasets used to reverse engineer them.

*Datasets*				*Interactomes*		
*Tissue type*	*Samples*	*Platform*	*Ref*	*Regulator*	*Targets*	*Interactions*
*Breast carcinoma (brca)*	1,037	RNAseq	TCGA	6054	19359	331919
*B-cell (bcell)*	201	HU133	Basso, K. et al. [23]	3537	13108	2005179
*Colon adenocarcinoma (coad)*	434	RNAseq	TCGA	6056	19820	413789
*Kidney renal clear cell carcinoma (kirc)*	506	RNAseq	TCGA	6054	19843	350478
*Lung adenocarcinoma (luad)*	488	RNAseq	TCGA	6055	19742	399513
*Prostate adenocarcinoma (prad)*	297	RNAseq	TCGA	6053	19820	33092
*Liver hepatocellular carcinoma (lihc)*	166	RNAseq	TCGA	6056	19829	469922
*Skin cutaneous melanoma (skcm)*	82	RNAseq	TCGA	5932	20531	877010
*Acute myeloid leukemia (laml)*	173	RNAseq	TCGA	6007	19269	531535
*String network*	NA	NA	Szklarczyk, D. et al. [24]	5662	12264	246596
*CHEA network*	NA	NA	Lachmann, A. *et al*. [25]	105	33009	196484
*Gene KD network*	NA	NA	Barrett, T. et al. [26]	650	23940	260000

**Fig 1:**
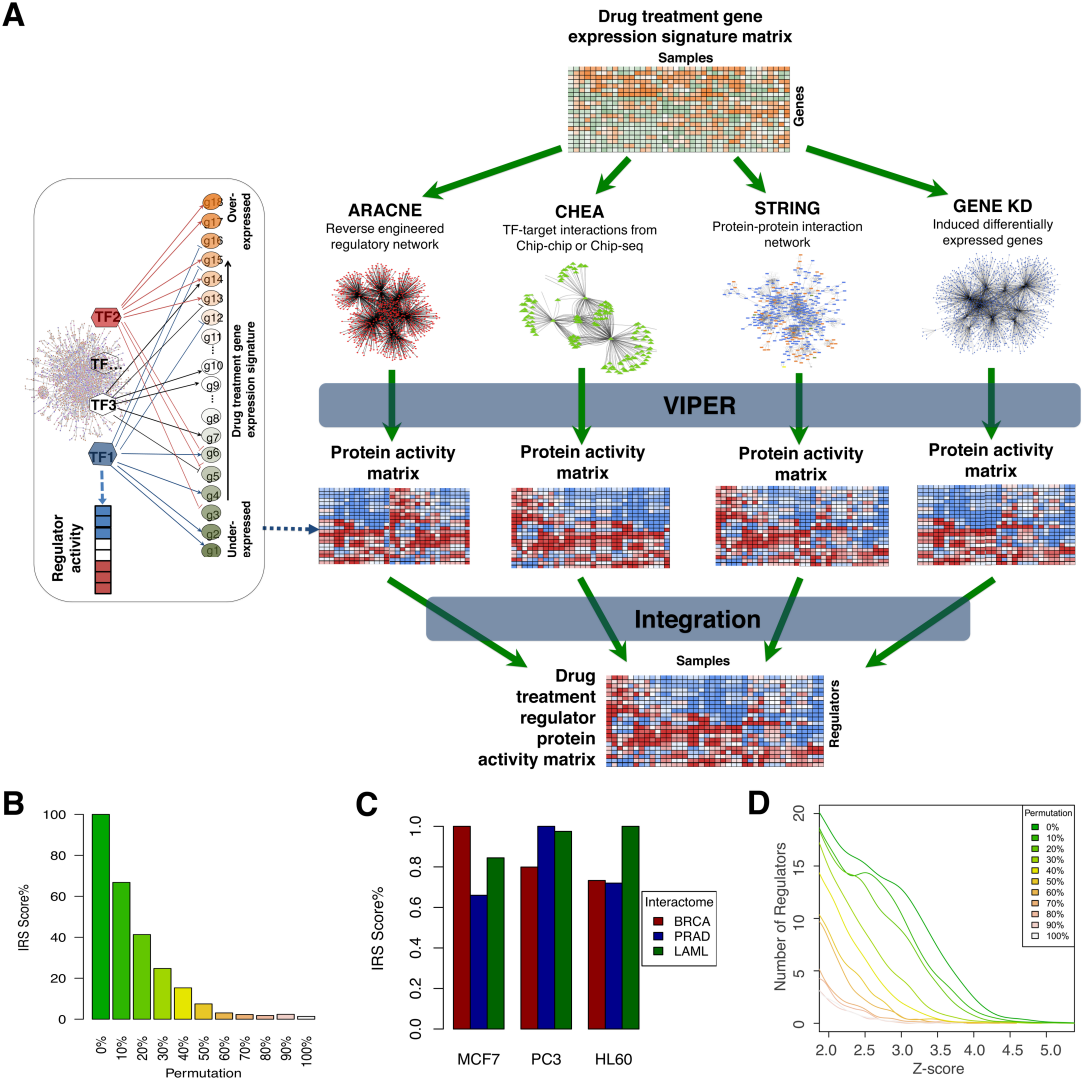
OncoLead: Network-based protein activity inference. (A) Drug perturbation induced genome-wide transcriptional changes are interpreted, based on multiple networks including ARACNE network, CHEA network, STRING network, and Gene knock-down (KD) network, with the VIPER algorithm, to infer changes in the activities of the regulatory proteins. The resulting four different protein activity matrixes were integrated into a single final protein activity matrix. In this way, VIPER analysis transforms drug-perturbation gene expression signatures into unbiased genome-wide regulator protein activity representations of CMoA. The left part is a simple illustration of how VIPER algorithm works based on the ARACNE network. First, ARACNE reverse engineers context-specific regulatory networks by leveraging a large collection of gene expression profiles (N > 100) from the same cellular context. Then, regulator’s activity is inferred by computing the enrichment of the genes in its regulon (from ARACNE) in every drug treatment signature sorted from the most over-expressed (colored in orange) to the most under-expressed (colored in green) genes. When there is positive or negative enrichment, the regulator is up-regulated or down-regulated (colored in red/blue). Regulator’s activity is represented by the normalized enrichment score. (B) IRS score decreases when progressively degrading the networks for MCF7 drug signatures. (C) Relative representation of how accurate each interactome is as a model for the transcriptional regulation in each of the three cell lines MCF7, PC3, and HL60 in CMAP database. Shown is the IRS in relative units for TRs inferred by OncoLead on each interactome (x-axis) / GES combination (see Methods for details). Percent IRS scores were obtained by dividing each specific IRS score by the largest score obtained across the three interactomes used in the analysis. (D) Distribution of the significant TRs inferred by OncoLead when adding increasing ratios of random noises to the Irinotecan signature in MCF7 cell line.

We further complemented the ARACNE networks by incorporating evidences from other resources, including direct TF to target genes interaction evidences from chip-seq or chip-chip data (ChEA database) [25], direct or indirect protein-protein interactions from the STRING database [24], and indirect functional associations inferred upon RNAi-mediated gene silencing experiments collected from the GEO database [26] (see Methods). Integration of these different evidences was performed at the inferred protein activity level (see Methods and Fig 1A).

To quantitatively assess interactome quality, we computed the Interactome Reliability Scores (IRS) as the area under the curve (AUC) representing the number of statistically significant OncoLead-inferred CMoA proteins as a function of the *p*-value threshold (see Methods). The rationale, as previously discussed [10], is that less accurate interactome models yield fewer statistically significant proteins, and thus lower IRS than the more accurate ones. Indeed, IRS scores decreased monotonically when protein interactions were increasingly randomized (0%–100%) using a degree-preserving randomization algorithm [27] (Fig 1B). Furthermore, confirming our hypothesis, tissue-matched interactomes systematically achieved the best IRS performance against the corresponding cell line specific signatures (Fig 1C).

Gene expression signatures (GES) representing each cell line following compound’s perturbations were then analyzed using OncoLead on multiple networks to generate integrated results. This produced a sparse 3-dimensional matrix of protein activity signatures [**ΔA**_*P*,*L*,*C*_], representing the relative differential activity (treatment with compound vs. DMSO control) of each target protein, *P*, expressed as Normalized Enrichment Score (NES), in cell line *L*, with compound *C*. This matrix thus provides a quantitative representation of the CMoA of all tested compounds, across all profiled cell lines (S1 and S2 Tables).

### CMoA proteins include established compound’s targets

#### Analysis of CMAP database

We systematically evaluated all CMAP profiled compounds that were reported in DrugBank [28] as specific agonists or antagonists (henceforth *modulators*) of any protein whose differential activity could be assessed by OncoLead analysis (Methods). Of 3,095 CMAP profiled samples in the MCF7 cell line, 198 had consistent gene expression signatures among replicate samples and were reported in DrugBank [28] as specific modulators of 75 distinct TFs or SIGs expressed in MCF7, such as *ESR1*, *TOP2A*, *NFKB1*, and *CDK2*, among the ~7,000 regulatory proteins that could be analyzed by OncoLead.

**Fig 2:**
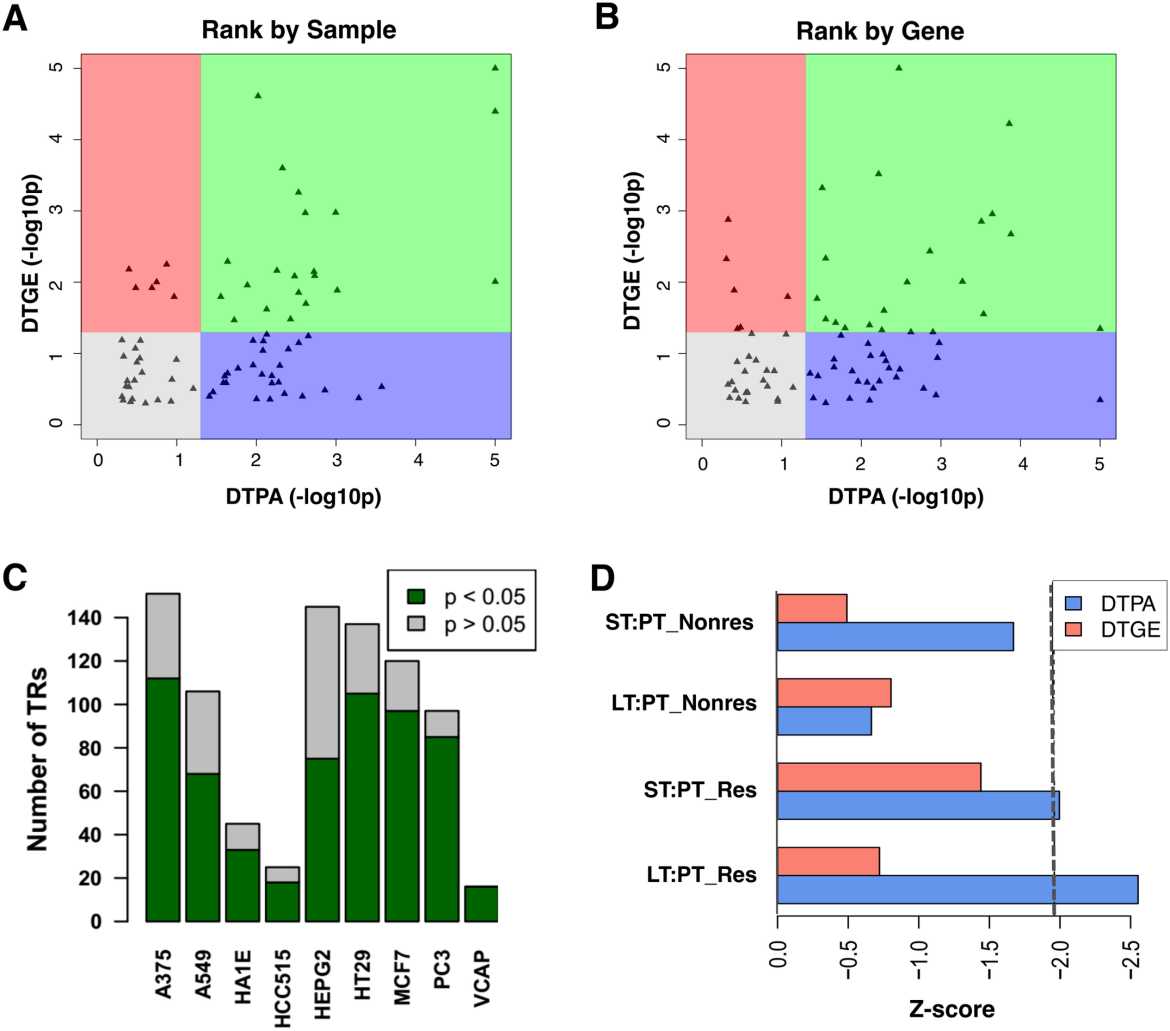
CMAP and LINCS dataset analysis. (A—B) Analysis for drugs targeting 75 TRs using either DTPA or DTGE for CMAP MCF7 datasets. For each TR with known inhibitors in the MCF7 dataset, we performed gene set enrichment analysis to test whether its DTPA or DTGE for its known inhibitors are significantly more inactivated or repressed compared to all other compound’s profiles (A) or to all other proteins (B) and obtained p-values from each test. Then we plotted the distributions of the–log10 p-values for DTPA (x-axis) versus DTGE (y-axis). Each triangle represents a TR. A vertical and a horizontal line were drawn at p-value equals 0.05 for DTPA and DTGE, respectively, which divide the plot into four parts: green, blue, red, and grey. (C) Enrichment analysis of the drug samples similar to TR silencing profiles on the vector of all drug samples in the same cell line sorted based on their inferred TR activity from LINCS data set. Each bar represents a cell line. Green color shows the number of TRs with significant enrichment (NES > 1.96; p < 0.05) which indicates the correlation between OncoLead CMoA inference and shRNA mediated TR silencing. Grey color shows the number of TRs without significant enrichment (NES< 1.96; p > 0.05). (D) OncoLead-inferred *ESR1* activity changes (blue) and the differential ESR1 expression (red) upon letrozole treatment *in vivo*. Signatures are obtained by comparing different time points in responsive and non-responsive patients. The values shown in the figures are Z-scores based on p-value. The black dotted line represents Z-score = 1.96 and p-value = 0.05.

To evaluate the value of OncoLead analysis in assessing the activity of compounds on candidate target proteins we performed two analyses. First, we asked whether established pharmacological modulators of a given target protein induced significant differential target protein activity (DTPA) (by OncoLead analysis) and differential target gene expression (DTGE) compared to all other compounds, see Fig 2A, S1A and S1C Fig. As shown, samples representing treatment with target-specific antagonists or agonists of 48 targets were significantly enriched among those with the most significant DTPA, compared to 27 enriched targets by DTGE (p < 0.05). Overall, DTPA outperformed DTGE in 41/75 targets (55%), including high therapeutic value proteins, such as *ESR1*, *NF-κB*, *NR3C1*, *PGR*, *HDAC*, *TOP2*, and *MTOR*, while DTGE outperformed DTPA in only 13/75 (17%) targets. Then we asked the reverse question. Specifically, whether established targets of a tested compound had statistically significant DTPA or DTGE compared to all other proteins, including all ~7,000 TF and SIG proteins evaluated in the study. (Fig 2B, S1B and S1D Fig). Again, DTPA outperformed DTGE for 42/75 (56%) target proteins, and DTGE outperformed DTPA for 12/75 (16%) targets. More specifically, proteins with the most significant DTPA were significantly enriched in 48 established compound’s targets (p < 0.05). While proteins with the most significant DTGE were enriched in only 26 known compound’s targets (p < 0.05). We have to note however, that DTPA and DTGE constitute an over-conservative test, since we cannot rule out potential effects of the remaining compounds on the targeted protein of interest, or whether the tested compound also affect some of the other evaluated proteins not established as a specific targets (we will refer to these proteins-compounds as off-target effects). Overall, DTPA metric dramatically outperformed DTGE, when evaluated across all relevant target proteins, both in terms of assessing on-target activity of known pharmacological modulators as well as assessing modulation of established pharmacological targets. Similar results were obtained from PC3 and HL60 perturbations; see Supplementary S1 Fig, suggesting effectiveness across multiple cellular contexts.

In all metrics, ~35% of known compound-target relationships could not be effectively assessed by OncoLead analysis. This can happen for several reasons. For instance, target proteins may not be expressed or have relevant activity in the specific context in which the compound was profiled. Alternatively, the protein regulon may be inaccurate, or the compound’s polypharmacology may be significant resulting in a vast set of secondary effects involving other proteins. Finally, the compound may have been profiled at a concentration that is not relevant in terms of target inhibition. The latter is a key problem because CMAP compounds were profiled at predetermined concentrations not directly related to their potency. For instance, while methotrexate has an IC_50_ of 196 nM on MCF7 cells[29], it was tested at 8.8 uM in CMAP.

### Analysis of LINCS repositories

We then expanded this analysis by leveraging an extensive collection of gene expression profiles, representing treatment of multiple cell lines with various compounds and shRNAs targeting different genes, available from the LINCS repository (http://lincs.hms.harvard.edu/). These datasets provide a limited representation, restricted to only 978 reporter genes (L1000) measured by a multiplexed Luminex assay. Within the constraints of such reduced representation, we used this dataset to build an experimental gold standard dataset (GSD) of compounds affecting the activity of specific target genes, by matching the signature of compound’s perturbations to those of shRNA mediated silencing. We limited our analysis to 1,365 compounds yielding statistically reproducible transcriptional responses (see Methods), and 92 shRNA-mediated silencing assays for which (a) target gene silencing could be confirmed by L1000 measurements at >3 standard deviations from the controls mean and (b) the gene was represented in the interactome as regulator (i.e. RP). This resulted in distinct gene silencing assays for each cell line, from a minimum of 16 in VCAP prostate cancer cells to a maximum of 151 in A375 melanoma cells.

To assemble a suitable experimental GSD, compound’s perturbations were matched to gene-silencing assays by Pearson correlation analysis of the corresponding, cell-matched L1000 signatures. Thus, each of the 1,365 most reproducible compound’s perturbations were associated to a list of shRNA-mediated gene silencing assays, ranked from the one with the most correlated to the one with the most anti-correlated L1000 signature. The rationale is that the gene-silencing assays with signatures most correlated to a compound’s perturbation signature represent proteins whose activity is inhibited by the compound.

We then computed the DTPA of each target protein for each of the perturbed cell lines, using the full gene expression profile, from regression analysis of the L1000 signature, see Methods. Thus, for each target protein, we rank-sorted all compounds by DTPA score, from its strongest predicted candidate inhibitor (i.e., that with the largest negative DTPA) to its strongest activator (*i*.*e*., that with the largest positive DTPA). Finally, we assessed these predictions by reciprocal gene set enrichment analysis (GSEA)[30] of the OncoLead-predictions against the experimentally-prioritized target modulators in the GSD. Specifically, for each target protein, we computed the NES representing enrichment of DTPA ranked inhibitors in statistically significant GSD inhibitors (*p* = 0.05).

Enrichment was statistically significant for most proteins targeted by small molecule compounds (NES > 1.96; p < 0.05, shown in green for RPs, Fig 2C and S2 Fig). This includes 112/151 proteins in A375 cells (74%), 68/106 in A549 cells (64%), 33/45 (73%) in HA1E cells, 19/25 (72%) in HCC515 cells, 75/145 (52%) in HEPG2 cells, 105/137 (52%) in HT29 cells, 97/120 (81%) in MCF7 cells, 85/97 (88%) in PC3 cells, and 16/16 (100%) in VCAP cells. Overall 609/842 testable proteins (72%) yielded OncoLead-inferred candidate inhibitors that were strongly enriched in experimentally assessed ones, based on the GSD. This is especially remarkable considering that LINCS L1000 assays directly measure expression of only 978 genes. As a result, on average, only 1/20^th^ of regulon targets is directly measured by these assays while other targets are imputed. In addition, shRNA-mediated silencing may have significant off-target effects. Taken together, these data suggest that the method represents an effective strategy to prioritize candidate inhibitors for arbitrary proteins of interest.

### Inferring compound’s MoA from patient-derived perturbations (*in vivo*)

To test whether OncoLead may be effective in elucidating the targets of specific compounds *in vivo*, we used gene expression data obtained from patient-derived tumor biopsies before and after therapeutic intervention. Specifically, we leveraged a dataset generated by Miller et.al (GSE20181)[31], consisting of primary breast tumor samples profiled after Letrozole treatment, including at 30-days (short term: ST) and 90-days (long-term LT), compared to pre-treatment profiles (PT). Letrozole blocks estrogen synthesis in postmenopausal patients by inhibiting the aromatase enzyme. This abrogates estrogen receptor activation in breast cancer cells. Individuals profiled in this dataset include 36 estrogen deprivation responsive and 14 non-responsive patients. Response was assessed based on whether significant tumor size reduction was observed at 90-days post-treatment.

Four differential expression signatures were analyzed, including ST:PT (30-days VS. pre-treatment) and LT:PT (90-days VS. pre-treatment), across both responsive and non-responsive patients. DTPA vectors were obtained by OncoLead analysis of these signatures, using the TCGA patient-derived Breast Carcinoma interactome (Table 1). As expected, *ESR1* activity was significantly reduced following Letrozole treatment in the responsive group (Fig 2D), with longer treatment inducing stronger *ESR1* activity reduction (*p*_LT:PT_ = 0.01; *p*_ST:PT_ = 0.046). Strikingly, however, OncoLead-inferred *ESR1* activity was not significantly affected by Letrozole in non-responsive patients (*p*_LT:PT_ = 0.51; *p*_ST:PT_ = 0.095). Furthermore, differential *ESR1* expression was not statistically significant following Letrozole treatment (p-value > 0.05, by Student’s t-test) at either time point and for either responsive or non-responsive patients, suggesting that CMoA analysis correctly captured *ESR1* inhibition even though its expression levels were not affected (Fig 2D).

### OncoLead-inferred CMoA provides a metric for context specific compound’s bioactivity

Since the IRS of each perturbation summarizes the effect of such perturbation on the inferred activity of regulatory proteins, we decided to use the IRS as a metric for the bioactivity of the small molecule compound. Specifically, we evaluated the IRS score across all cell lines and compound’s perturbations in the Connectivity Map (CMAP) dataset. As expected, progressive degradation of the gene expression signatures, by randomly permuting increasingly larger subsets of gene expression values, was associated with a proportional decrease in the IRS (Fig 1D). We selected Irinotecan for this test because it showed one of the highest IRS values in CMAP.

Among the 1,294 CMAP compounds, HDAC, topoisomerase, CDK, and estrogen receptor antagonists presented the largest overall IRS in the MCF7 luminal breast cancer cell line. These compounds represent well-known cancer drug classes, currently under investigation in breast cancer clinical trials, and thus likely to be highly bioactive in these cells. The same analysis performed on the other two CMAP cell lines (*i*.*e*., PC3 and HL60), consistently identified HDAC, HSP90, NF-KB, topoisomerase, proteasome and protein synthesis inhibitors among the compounds with highest IRS. Interestingly, we observed a low IRS for a large proportion (~55%) of the compounds profiled in MCF7 cells in CMAP, suggesting poor bioactivity of those compounds at the profiled concentrations.

The specific highest IRS in MCF7 (breast cancer, BRCA), HL60 (acute promyelocytic leukemia, APL), and PC3 (prostate cancer, PRAD) cells was achieved by fulvestrant (*ESR1* antagonist), tretinoin (all-trans retinoic acid), and pioglitazone, respectively. Estrogen antagonists represent the standard of care in BRCA adjuvant therapy[32]. Indeed, based on NCI60 data[33], fulvestrant achieves 50% growth inhibition (GI_50_) at a substantially lower concentration in MCF7 compared to HL60 (-LogGI_50_ (M) = 8 vs. 5.2). Similarly, all-trans retinoic acid represents the standard of care in APL[34]. Indeed, based on drug sensitivity profile in COSMIC data, HL60 cells were more sensitive to tretinoin than MCF7 and PC3 (LogIC_50_ (uM) = 0.83, 4.0 and 5.8 for HL60, MCF7, and PC3, respectively). Finally, pioglitazone, a PPAR-γ agonist approved by the FDA as anti-diabetes drug, showed higher IRS in PC3 compared to the other two cell lines. Interestingly, PPAR-γ agonists are currently in phase 2 clinical trials for AR-independent prostate cancer[35].

#### Validation of novel OncoLead-inferred transcriptional regulators (TRs)-inhibitors

Due to their direct effect on the cell transcriptional response, TRs are at the center of the machinery that integrates exogenous and endogenous signals to control physiologic and pathologic cell states. We have shown that master regulator (MR) TRs responsible for pathological transitions, can be systematically elucidated by differential activity analysis but not by differential expression[10, 17]. These MR TRs constitute key non-oncogene tumor dependencies, eliciting either essentiality or synthetic lethality in vitro and in vivo[8, 10]. Unfortunately, TRs are considered to be “undruggable” targets because the DNA-interacting surface is highly charged resulting in unfavorable drug target properties[36].

We thus tested whether OncoLead may be used to reposition compounds profiled in CMAP as context-specific TR inhibitors. We have generated a comprehensive map of predicted compound-TR interactions in the MCF7 breast carcinoma cell context (S1 and S2 Tables). We selected *MYC* and *STAT3* oncogenes for further experimental validation, because of their critical role in tumorigenesis[37, 38] and availability of suitable luciferase-based gene reporter assays to assess their transcriptional activity (see Methods for details).

**Fig 3:**
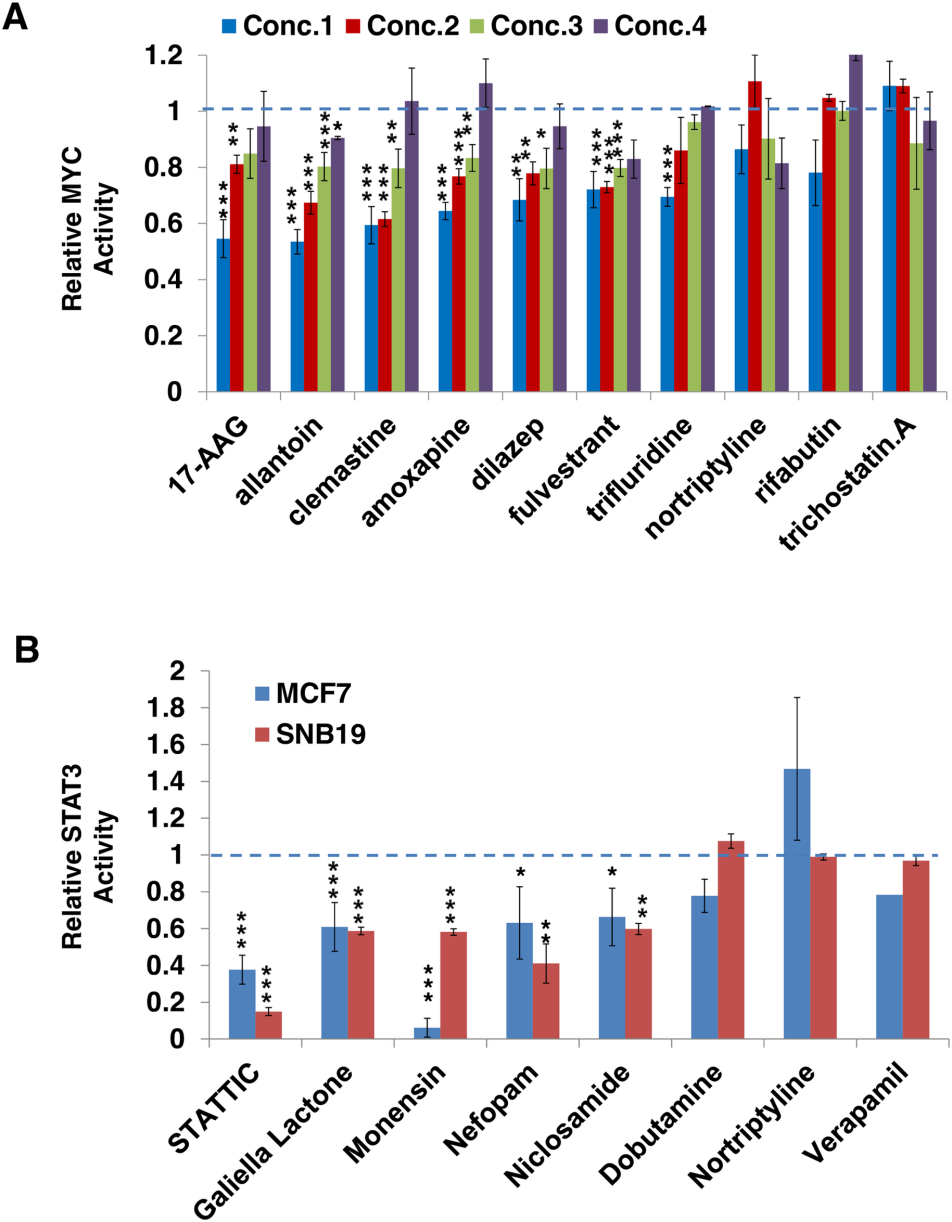
Validation of predicted novel modulators for MYC and STAT3. (A) Effect of computationally predicted drug modulators on *MYC* activity in luciferase assay. The compounds were tested by *MYC* luciferase reporter assay in MCF7 cell line. Concentrations for each compound are shown in S4 Table. The reporter activity for each compound was normalized to cell titer and compared to DMSO control. We compared drug induced activity vs. DMSO control using t-test. (p< 0.01: ***; p < 0.05: **; p<0.1: *) (B) *STAT3* luciferase reporter assay results in MCF7 (blue) and SNB19 (red). The compounds were tested by *STAT3* luciferase reporter assay in MCF7 and SNB19 cell line. Each compound was tested at 10uM. The activity for each drug is computed using the reporter activity normalized to cell titer and compared to DMSO control. We compared drug induced activity vs. DMSO control using t-test. (p< 0.01: ***; p < 0.05: **; p<0.1: *).

We first selected ten compounds showing the highest OncoLead-inferred *MYC* inhibitor activity (S3 Table). To minimize the impact of drug induced cell death on luciferase reporter assays, we used sub-lethal concentrations for the compound, starting at the IC_20_ at 48h, as assessed by ATP viability assays, and at three additional serial dilutions (S4 Table). Seven of the ten predicted candidate *MYC* inhibitors showed a dose-dependent reduction in *MYC* reporter signal (Fig 3A and S4 Table), including 17-AAG, allantoin, amoxapine, clemastine, dilazep, fulvestrant and trifluridine. Interestingly, fulvestrant (500nM), an *ESR1* antagonist, was previously shown to decrease *MYC* protein levels in MCF7 cells[39], and thus represents a positive control in our assay. In another study, 17-AAG (an *HSP90* inhibitor) was reported as an inhibitor of *STK38* [40] and we have characterized *STK38* as an extremely potent post-translational modulator of *MYC* turnover[41], thus mechanistically supporting 17-AAG mediated inhibition of *MYC* transcriptional activity in this study.

*STAT3* has been reported as a proto-oncogene in several human cancers, suggesting significant potential for targeted therapy[42]. We tested eight compounds, including six inferred as the most significant *STAT3* inhibitors by OncoLead analysis of CMap data (niclosamide, monensin, nefopam, nortriptyline, dobutamine and verapamil) (S3 Table), and two commercially available *STAT3* inhibitors as positive controls (Stattic and Galielolactone)[43, 44]; see Methods. Three of the six predicted compounds, including niclosamide, monensin, and nefopam, significantly affected the *STAT3* gene reporter assay signal in MCF7 cells, with effects comparable to the positive controls (Fig 3B).

Finally, to assess whether OncoLead-inferred target inhibitors are conserved across different cellular contexts, we tested the breast cancer specific prediction for *STAT3* inhibitors in human glioblastoma cells. Specifically, we first generated SNB19 human glioma cells stably transduced with the *STAT3* gene reporter assay originally developed for MCF7 cells (Fig 3B). The effect on *STAT3* activity was fully recapitulated in SNB19 cells, supporting our previous observations that OncoLead inferred CMoA proteins are conserved across distinct cellular contexts.

Among the three validated *STAT3* inhibitors, niclosamide was previously reported as a *STAT3* inhibitor[45]. There also have been controversial studies regarding whether monensin inhibits *STAT3* activity[46–48]. We show that monensin is a strong *STAT3* inhibitor both in MCF7 and SNB19 cells, and report nefopam as a novel *STAT3* inhibitor.

#### TR DTPA signature provides a compact representation of the cell state induced by the compound

**Fig 4:**
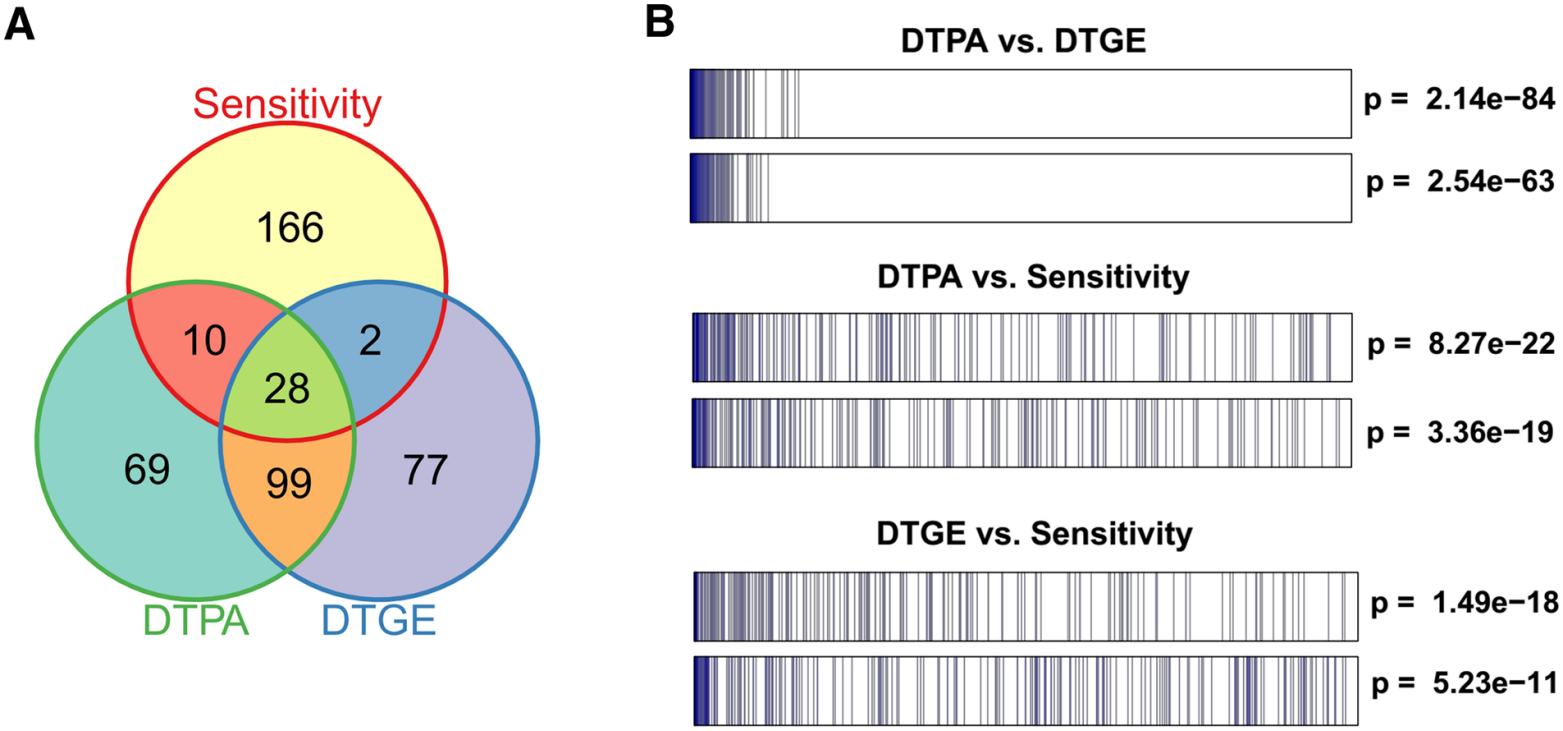
Comparison of CMoA, GES and GI_50_ profile similarities. (A) Venn diagram of the top 206 similar compound pairs (top 2.5%) using DTPA, DTGE and GI_50_ sensitivity profiles. (B) top: Enrichment of the top 206 pairs based on DTPA similarity in the vector of 8256 (129*128/2) compound pairs ranked by DTGE similarity, and vice versa; middle: Enrichment of the top 206 pairs based on DTPA similarity in the vector of 8256 (129*128/2) compound pairs ranked by GI_50_ correlation, and vice versa; bottom: Enrichment plot of the top 206 pairs using DTGE similarity in the vector of 8256 (129*128/2) compound pairs ranked by GI_50_ correlation, and vice versa.

Given a sufficiently large number of distinct cell lines, CMoA similarity is effectively captured by the similarity in their panel-specific sensitivity profile[49, 50]. We thus leveraged the NCI60[33] dataset, which contains GI_50_ profiles for ~30,000 compounds across 60 cancer cell lines representing different tissue lineages, including 129 compounds profiled at the gene expression level in the MCF7, PC3 and HL60 CMAP datasets. The Spearman correlation of the GI_50_ values across all tested cell lines was used as an objective compound’s similarity metric. Comparing the 2.5% most similar pairs selected either based on chemosensitivity, CMoA, or GES based similarity produced remarkable overlap (p < 2.2e-16 or p = 10^−14^, by Fisher’s Exact Test, FET; Fig 4A). These results were recapitulated by enrichment analysis of the top 2.5% most similar pairs identified by each approach on the ranked lists by the other approaches (Fig 4B). For this analysis, we averaged the GES and CMoA similarity scores across the three cell lines profiled by CMAP.

These results show that despite a 20-fold more compact representation, CMoA signatures are as reliable as GES signatures for assessing compound’s MoA similarity. In addition, our data shows that CMoA is much more reproducible than GES, especially for single-sample analysis. Specifically, CMoA signatures representing replicated CMAP drug-perturbed samples were significantly more correlated (by Spearman correlation) than GES signatures (S3 Fig). The reason is two-fold: First, OncoLead infers differential activity of a protein by integrating the differential gene expression of all of its regulon genes, instead of relying on a single mRNA abundance measurement. Second, the regulatory model helps integrate the biological component of the gene expression variance while effectively filtering technical noise contributions, as they are not compatible with the model. Taken together, this makes CMoA highly resilient to the noise typical of gene expression measurements[20]. Moreover, since the regulatory models account for tissue specific regulation, CMoA profiles were significantly more correlated than corresponding GES profiles across cell lines (S3 Fig). This helps integrate mechanism of action evidence across distinct cellular contexts.

### A systematic regulatory footprint of compound’s perturbations

Our analysis provides a comprehensive map of transcription regulator (TR), whose activity is modulated by a large repertoire of compounds across the CMAP and LINCS perturbational databases. This represents a large set of cellular contexts, including: MCF7 (breast), PC3 (prostate), HL60 (leukemia) in CMAP, and A375 (skin), A549 (lung), HA1E (kidney), HCC515 (lung), HEPG2 (liver), HT29 (colon), MCF7 (breast), PC3 (prostate), and VCAP (prostate) in LINCS (S1 and S2 Tables). Specifically, a compound-TR activity matrix was generated for each cell line, with rows representing TR proteins and columns representing compound’s perturbations. This matrix can be used to perform a variety of analyses, including identifying optimal context-specific compounds to inhibit or activate an arbitrary TR protein, as well as to compute the similarity between compounds.

## Discussion

Traditional drug discovery has been focused on one-drug / one-target strategy. More recently, the approach has been expanded to poly-pharmacological effects of the drugs, meaning that one drug can interact with multiple on-target and off-targets resulting in combination of wanted and unwanted effects [51]. However, MoA discovery is still limited to compound’s direct binders [4, 5]. In this work, we expand the concept of drug MoA by including direct and indirect mediators of compound’s effect. We limit our approach however, to proteins having a transcriptional regulatory role in the cell (TR). Given their central role as regulators of cell state [10, 17], limiting CMoA definition to TRs dramatically reduce the dimensionality of the problem with no reduction of sensitivity to detect compound’s biological activity. Due to signaling and transcriptional network rewiring, inclusion of non-direct targets in CMoA makes the approach exquisitely cell context specific.

While small molecule compounds affect mostly the activity of the directly or indirectly targeted proteins, we currently have no methods in our biochemistry arsenal to directly measure protein activity. While important advances in the field of proteomics have made possible the quantification of proteins and protein isoforms in a close-to proteome-wide fashion, protein activity is not solely determined by protein or protein isoform abundance, but also depends on proper cellular localization and interaction with co-factors. Gene expression, on the other hand, can be actually profiled with high accuracy and at relative low cost, making it one of the phenotypic read-outs of choice for current drug-screen efforts. mRNA abundance however, is not directly associated with coded protein activity, especially after compound-mediated short-term perturbations, but can be interpreted, using context-specific models of transcriptional regulation, to infer changes in the regulatory proteins activity [10].

At the core of our approach is the VIPER algorithm [10, 18], which makes possible several fundamental characteristics: (1) the analysis can be performed at the single-sample level, allowing its application to FDA-approved drug repositioning in cancer precision medicine protocols (see below), (2) results are very robust to regulatory model accuracy and expression profile quality, and (3) results are almost insensitive to partial transcriptome coverage, making it particularly suited for the analysis of low-depth RNAseq expression profiles following drug perturbation [18]. In fact, we have shown that VIPER-activity signatures obtained from 1 million reads per sample are virtually identical to the ones obtained from 30 million reads, while GES were dramatically different. This remarkable quality enables us to infer drug MoA from ultra-low cost high-multiplex expression profile analysis following drug perturbation. VIPER can accurately identify regulators whose activity is modulated by the compound. However, in ~10% of the cases, it may switch the effect directionality (e.g., infer a protein as activated when is in fact inhibited) [20]. This occurs because, at steady state, auto regulatory loops may exist that induce inverse correlation between protein activity and mRNA expression. As a result, targets may be correctly inferred but their relationship (activated vs. repressed targets) may be inverted.

We recently introduced DeMAND, a method for the elucidation of compound MoAs, based on compound’s perturbational gene expression profiles[13]. Although OncoLead and DeMAND are both network-based, they are fundamentally different and complementary, both in their formulation and, more importantly, in their practical applications. More specifically, while DeMAND focuses on compound-mediated dysregulation of protein interactions, thus requiring at least six distinct perturbations for reliable predictions, OncoLead directly infers changes in protein activity based on their regulon differential expression, thus requiring a single sample. Using the same benchmarks that were used to evaluate performance of the DeMAND algorithm, the perturbational profiles of fourteen compounds in LY3, we tested the algorithm complementarity of the OncoLead and the DeMAND. OncoLead outperformed DeMAND almost by the same margin by which DeMAND outperformed a naïve t-test analysis, as reported in[13] (S4A Fig). When integrating the predictions from the two methods, each protein was assigned the best of the OncoLead or DeMAND scores. The integrated results outperformed both individual method predictions, confirming the significant complementarity of these methods (S4A Fig). For example, while DeMAND missed the direct doxorubicin target (*TOP2A*), this was effectively captured by OncoLead (ranked 25 out of 6,819 targets, *p* = 4.7x10^-22^). Conversely, while OncoLead missed geldanamycin’s target HSP90AA1, it was effectively discovered by DeMAND (ranked 6 out of 7499 targets, *p* = 1x10^-25^). In general, OncoLead is better suited to analyzing proteins that have a direct regulatory role, while DeMAND is more effective on proteins involved in non-regulatory interactions, such as those involved in heat stress responses. As a result, the integration of both methods is optimally suited for the elucidation of compound’s MoA.

A clear limitation of our approach however, is that the definition of drug MoA is limited to the proteins represented as regulators in the network models. This leaves out non-protein mediators of drug effect as well as proteins not considered as regulators (TRs in this manuscript). However, because any drug-induced phenotypic change reflected in transcriptome changes will be partially mediated by transcriptional regulators, genome-wide coverage of TRs by the regulatory models should be enough to capture, at least partially, the MoA of any bioactive compound.

This study presents a new framework for defining compound’s MoA. We have shown that OncoLead-MoA captures the direct targets for known drugs while GES does not. Moreover, by interpreting drug-induced GES with models of transcriptional regulation, OncoLead-MoA is insensitive to signature noise while providing information about the bioactivity of the compounds, even when no replicated profiles are available. This is because non-informative signatures, generated from non-bioactive compounds exposure, will be poorly explained by the regulatory models and hence, their IRS scores will tend to be small, compared to informative, bioactive compound-derived expression signatures (S4B Fig).

We envision a strong impact of our approach for drug repositioning in precision medicine. Specifically, our method can be applied to infer drugs targeting patient-specific and currently ‘undruggable’ targets. For instance, we have shown that tumor subtype-specific MRs constitute tumor dependencies[8–10], which are usually non-tractable from the current repertoire of FDA-approved drugs. However, inhibitors for such MRs can be inferred from gene expression data of appropriate models after drug perturbation, as we have shown here for *MYC* and *STAT3*. We can envision a framework for cancer precision medicine in which we first infer the MRs for a single-patient in an unbias genome-wide fashion. Then by matching the patient’s MR profile with the full FDA-approved drug OncoLead-MoA, in the spirit of the Connectivity Map [11], we select drugs or drugs combinations that not only target the top patient MR, but that comprehensively target a significant proportion of them. The elevated noise in single-tumor gene expression profiles makes this approach to be unfeasible if based only on gene expression. Conversely, interpretation of the signatures based on regulatory models, which are based on tenth to hundreds of genes per protein regulon, makes OncoLead results highly reproducible [20], and the single-patient MR signatures, as well as the single-sample based drug MoA obtained from drug-screen experiments, extremely robust (S4C Fig). If MRs are enriched in tumor dependencies [9, 10], then this approach should prioritize drugs being specifically toxic for the patient’s tumor.

## Materials and methods

### OncoLead

#### Constructing context-specific regulatory network using ARACNE

We applied the ARACNE algorithm to reverse engineer the regulatory networks from different datasets. The tissue networks were reverse engineered from the RNASEQ samples in TCGA databases for different lineages including breast (brca), prostate (prad), leukemia (laml), colon (coad), kidney (kirc), lung (luad), liver (lihc) and skin (skcm). We downloaded the level 3 RNASEQ data from TCGA. The raw counts were normalized and the variance was stabilized with the DESeq R package [52]. The B-cell tissue interactome were built from HG-U133plus2 affymetrix microarray samples. The Affymetrix platform data sets were normalized using MAS5 algorithm. 1877 genes annotated as transcription factors and 3555 genes annotated as signal transduction in Gene Ontology Database were included in the ARACNE run. ARACNe was run with 100 bootstrap steps using DPI (data processing inequality) tolerance threshold set to 0 and MI (mutual information) p-value cutoff set to 10E-7. Regulatory networks used in this study are deposited in figshare and could be downloaded from https://figshare.com/articles/Regulatory_networks_reverse_engineered_from_gene_expression_profiles_of_cancer_tissues/4742209. ARACNE software is available from the authors’ website (http://califano.c2b2.columbia.edu/aracne).

#### Constructing protein-protein interaction network from the STRING database [24]

The protein-protein interactions were downloaded from the STRING database, where the interactions with confidence score larger than 0.7 were kept. For each protein, all its interacting proteins were considered as its “regulon” for the subsequent OncoLead analysis.

#### Constructing the TF regulatory network from the CHEA database[25]

The TF-target genes were obtained from the CHEA database based on the Chip-chip or chip-seq data from the ENCODE project. We downloaded data where the experiments were performed in human cells. For multiple experiments involving the same TF genes, we used all the identified target genes from multiple experiments as the “regulon” for the particular TF.

#### Constructing signaling network based on gene knock-down experiments

The gene knock-down expression profiles were obtained from the GEO database. We downloaded all GEO files where their “summary” or “title” or “overall_design” sections contain patterns like “knockdown”, “knock-down”, “knock down”, “shRNA”, “siRNA”, “RNAi”, “hairpin”, “silence”, or “silencing”. Then, for each file, the knock-down signatures were generated using t-test in Limma package by comparing the knock-down versus the control samples. For each experiment, we first checked the knock-down efficiency and kept only signatures where the genes that were silenced had p-value less than 0.1 based on the t-test. For multiple signatures targeting the same gene, we first convert the p-values to z-scores for each gene, and then used Stouffer’s method to generate the integrated signature.

#### Generating gene expression signatures after compound’s treatment

We downloaded the data matrix from the CMAP built 2. We map the probes to the same gene and keep only one probe for each gene with the maximum median deviation (MAD) score across all samples. The drug signature is obtained by scale each gene across all the samples for each cell line. We obtain a random signature for the CMAP-MCF7 data set by running t-test comparing the gene expression profiles of 100 randomly selected MCF7 samples with another 100 randomly selected MCF7 samples and computed the -log (p-value,10) for each gene. We run this for 1000 times to obtain the null model for MCF7 drug signatures. Similar process was performed in PC3 and HL60 in CMAP database. For OncoLead analysis, we kept only the drugs that have similar replicate samples for each cell line.

The library of integrated network-based cellular signatures (LINCS) is a dataset of gene expression signatures derived from a variety of cell lines. Cells are treated with chemical compounds or are subject to gene knockdown and overexpression. It is the largest Gene Expression High Throughput Screening (GE-HTS) to date and is an extension of the Connectivity Map (CMAP) experiment. In contrast to most GE-HTS datasets for the LINCS project a Luminex bead assay was used to measure the expression of about 1000 landmark genes (L1000). The gene expression of the remaining 22000 genes was estimated using a full liner model that was derived from 100000 gene expression profiles from the Gene Expression Omnibus (GEO). The L1000 genes are chosen to maintain most of the original information and fulfill the requirements of being minimally redundant, expressed widely through different cellular contexts and allow the inference of other genes outside the L1000 set. The L1000 data is first normalized by invariant set scaling of 80 invariant genes and then quantile normalized. The experiments are performed on 384 well plates. To compute signatures, we apply a z-score transform over the samples for each individual plate. The LINCS data is organized in batches of replicate plates. We calculate the reproducibility score for each sample in a batch by the average correlation to the replicate samples on the other plates. Only 72714 samples have highly significant reproducibility with z-score bigger than 5, from where 1,365 compound’s treatment signatures and 92 shRNA mediated silencing signatures were selected for further analysis.

For the letrozole treatment primary breast tumor data set, we downloaded the GSE20181[31] from GEO dataset and normalized this data set using RMA. To obtain the drug signatures, we ran t-test comparing gene expression profiles from different groups of samples. The null model was generated by randomly shuffling the labels of two groups of samples for 1000 times and ran t-test comparing the two groups to obtain the 1000 random signatures.

#### OncoLead analysis on different drug treatment signatures

OncoLead leverages the VIPER algorithm, which integrates the Mode of Regulation information, regulator-target interaction confidence and pleiotropy index for each regulon engineered from ARACNE algorithm[10]. Then 3-tail weighted rank based enrichment analysis is applied to each sample. The protein activity level changes for each sample are inferred and represented as p-value or NES (normalized enrichment score) which are computed using the null model generated for each data set. VIPER R-package is available from Bioconductor (https://www.bioconductor.org/packages/release/bioc/html/viper.html).

Based on each of the four networks (i.e., ARACNE, CHEA, STRING, and Gene KD), we thus transformed the gene expression*drug sample matrix to the TR-activity (NES)*drug sample matrix by running VIPER using each network. To integrate the four protein activity matrixes, for each protein activity induced by every compound’s treatment, we computed the weighted average of the activity scores generated using different networks weighted by the absolute value of the scores.

Then we computed for each protein activity*sample matrix, the distribution of the–log (p-value) of all TRs with FDR-corrected p-value < 0.05 and computed an area under the curve score (IRS score) for this distribution. This IRS score represents the overall significant TR activity changes for a drug signature dataset discovered by OncoLead.

### Gene set enrichment analysis (GSEA)

GSEA uses the Kolmogorov-Smirnov statistic and tests the enrichment of a gene set on a gene signature generated by t-test, fold-change or other methods[30, 53].

### Drug-drug distance

The distance between drugs based on gene expression or CMoA profiles was computed using signature distance algorithm. It takes the 5% most up-regulated and down-regulated genes (gene expression value or OncoLead activities) of one sample 1 (2) and computes its enrichment on the other sample 2 (1) to get value DIS1-2 (DIS2-1). Then take the average of DIS1-2 and DIS2-1 to get the distance between sample 1 and 2.

The distance between drugs based on GI50 sensitivity profiles from NCI60 was computed using spearman correlation of the sensitivity scores across different cell lines.

### Cell lines and Reagents

MCF7 and SNB19 cells were cultured in RPMI (Invitrogen) and supplemented with 10% FBS (Invitrogen) and 1% penicillin-streptomycin (Cellgro). Cell viability was measured using CellTiter-Fluor Cell Viability Assay (Promega, G6080). Cignal *STAT3* reporter (luc) kit was purchased from Quiagen (CCS-9028L). *MYC* reporter was bought from Qiagen/SABiosciences (CCS-012L). Reporter activity was determined by Dual-Glo Luciferase Assay System (Promega, E2920). Compounds were purchased from Sigma, Prestwick and Spectrum.

### MYC luciferase assay

MCF7 cells in RPMI 10% FBS with antibiotics were plated at the density of 2x10^4^ cell/well onto 96-well flat-bottom plate one day before transfection. Cells were transfected with *MYC* reporter using the jetPrime (Polyplus) delivery system. Reporter mixture contains an inducible *MYC* responsive firefly luciferase construct and constitutively express Renilla construct (ratio 40:1). A mixture of non-inducible firefly luciferase reporter constitutively expressing Renilla construct was used (ratio 40:1) as a negative control. Constructs constitutively expressing GFP, firefly luciferase and Renilla luciferase constructs (40:1:1) were used as positive control.

24 hours after cell seeding, culture medium was replaced with fresh medium (100 ul) and drugs were added to the cells in duplicates. 24 hours after drug treatment, cell viability and *MYC* reporter activity was measured. Serial dilutions of drugs were prepared in DMSO to keep the same final concentration of DMSO at 0.8% (S4 Table). DMSO only was used as a negative control for drugs. Gene reporter activity following drug treatment was normalized to cell viability (Firefly/CellTiter-Fluor) and compared to the negative controls (DMSO).

### STAT3 luciferase assay

MCF7 cells were first transfected with *STAT3* and stimulated using 40ng/ml IL6. 24 hrs later cells were treated with drugs at 10uM. For the control, corresponding amount of DMSO was added to the cells. 24hrs after the drug treatment, cell viability was determined and reporter activity was measured. Each experiment was performed in triplicate. The experimental procedure for SNB19 was the same for MCF7 except stimulation using IL6. Galliela Lactone and Static were included in the experiment as positive controls and DMSO was used as negative control.

## Supporting information

S1 Table OncoLead inferred CMAP—MCF7, PC3, and HL60 drug perturbational transcription regulator activity. Available from: https://figshare.com/s/635c0ee06b8b3448d12d
(PDF)

S2 Table OncoLead inferred LINCS—A375, A549, HA1E, HCC515, HEPG2, HT29, MCF7, PC3, and VCAP drug perturbational transcription regulator activity. Available from: https://figshare.com/s/09aaf3b437f47dff1eac
(PDF)

S3 Table Inferred MYC and STAT3 activities as well as differential gene expression values for the experimentally tested compounds. (XLSX)

S4 Table Selected concentrations for each compound around IC_20_ at 48h that are used in MYC reporter assay and summarized results of MYC single compound reporter assay. (XLSX)

S1 Fig (A-D) Analysis for drugs targeting 75TRs in PC3 (A-B) or 19 TRs in HL60 (C-D) using either DTPA or DTGE for CMAP datasets. (A, C) For each TR with known inhibitors in the PC3 or HL60 datasets, we performed gene set enrichment analysis to test whether its DTPA or DTGE for its known inhibitors are significantly more inactivated or repressed compared to all other compound’s profiles and obtained p-values from each test. Then we plotted the distributions of the–log10 p-values for DTPA (x-axis) versus DTGE (y-axis). Each triangle represents a TR. A vertical and a horizontal line were drawn at p-value equals 0.05 for DTPA and DTGE, respectively, which divide the plot into four parts: green, blue, red, and grey. (B, D) For each TR with known inhibitors in the PC3 or HL60 datasets, we performed gene set enrichment analysis to test whether its DTPA or DTGE for its known inhibitors are significantly more inactivated or repressed compared to all other proteins and obtained p-values from each test. Then we plotted the distributions of the–log10 p-values for DTPA (x-axis) versus DTGE (y-axis).
(TIF)

S2 Fig Enrichment analysis of the drug samples similar to TR silencing profiles on the vector of all drug samples in the same cell line sorted based on their inferred TR activity. Results are shown cell line by cell line. Each bar is the analysis for one TR. A dotted line is drawn at NES = 1.96 (p = 0.05). TRs with significant enrichment (NES > 1.96; p < 0.05) are colored in green indicating the correlation between OncoLead CMoA inference and shRNA mediated TR silencing. Grey color indicates no significant enrichment.
(TIF)

S3 Fig Boxplot of pearson correlation between the drug DTPA (blue) or DTGE (salmon) for the same drug replicates with the largest number of replicate samples, in the same cell lines (top panel) or across cell lines from different tissues from CMAP data set (bottom panel). (TIF)

S4 Fig (A) Boxplot of the AUC score (area under the ROC curves as a function of the top predictions for identifying the known targets in the Dream dataset) using either OncoLead (red), DEMAND (blue), T-TEST (green) or integrating OncoLead and DEMAND result (yellow). (B) Boxplot of IRS scores for drugs whose replicates are significantly similar to each other (N = 76) and drugs whose replicates are dissimilar to each other (N = 94). (C) Box plot of the ranking positions of the top 10 drugs selected from CMAP-MCF7 data based on DTPA (blue) or DTGE (salmon) distances to a luminal breast cancer sample signature when adding Gaussian noise to the signature. For this analysis, we randomly select one luminal breast cancer gene expression profile from TCGA data set and add different levels of Gaussian noise to this profile. The Gaussian noise is a normal distribution centered in zero with the same length as the length of the gene expression profile. We generated 20 different levels of Gaussian noise, each has a different standard deviation (SD) ranging from 10% to 200% of the SD of the original gene expression profile. Then, for each different SD, we produce 1000 random gaussian noises and add each of them to the original gene expression profile and get 1000 gene expression profiles. Then for these 1000 modified gene expression profiles as well as the original profile, we did z-score transformation by minus the mean and divided by standard deviation of the TCGA basal breast cancer samples for each gene and obtained 1001 DTPA signatures. After that, we run OncoLead on each signature using breast cancer interactome to get DTPA for each signature. To find drugs that best reversing these signatures, we compute pearson correlation between CMAP-MCF7 drug induced DTPA and the 1001 DTPA and between CMAP-MCF7 drug induced DTGE and the 1001 DTGE. Ten drugs are selected which have the largest negative correlation to the original patient DTPA or DTGE signature. Finally, we plot the ranking position distribution for each of these ten drugs based on the correlations using DTPA or DTGE signatures from the 1000 permutations. As we see, the ranking positions of the originally selected 10 drugs are much more preserved based on DTPA correlation even when there are large noises in the signatures, while they are much more sensitive based on DTGE correlation when there are increasing level of noises.
(TIF)
