# Supplementary material for: Systematic, network-based characterization of therapeutic target inhibitors
Source: PLoS Comput Biol. 2017 Oct 12;13(10):e1005599. doi: 10.1371/journal.pcbi.1005599 (PMC5638208; doi:10.1371/journal.pcbi.1005599)
Supplement: S1 Table — Available from: https://figshare.com/s/635c0ee06b8b3448d12d (PDF) [file pcbi.1005599.s001.pdf]

S1 Table: OncoLead inferred CMAP - MCF7, PC3, and HL60 drug perturbational transcription regulator activity. Available from: https://figshare.com/s/635c0ee06b8b3448d12d
