# Supplementary material for: Systematic, network-based characterization of therapeutic target inhibitors
Source: PLoS Comput Biol. 2017 Oct 12;13(10):e1005599. doi: 10.1371/journal.pcbi.1005599 (PMC5638208; doi:10.1371/journal.pcbi.1005599)
Supplement: S2 Table — Available from: https://figshare.com/s/09aaf3b437f47dff1eac (PDF) [file pcbi.1005599.s002.pdf]

S2 Table: OncoLead inferred LINCS - A375, A549, HA1E, HCC515, HEPG2, HT29, MCF7, PC3, and VCAP drug perturbational transcription regulator activity. Available from: https://figshare.com/s/09aaf3b437f47dff1eac
