# Supplementary figures and images for: Systematic, network-based characterization of therapeutic target inhibitors
Source: PLoS Comput Biol. 2017 Oct 12;13(10):e1005599. doi: 10.1371/journal.pcbi.1005599 (PMC5638208; doi:10.1371/journal.pcbi.1005599)

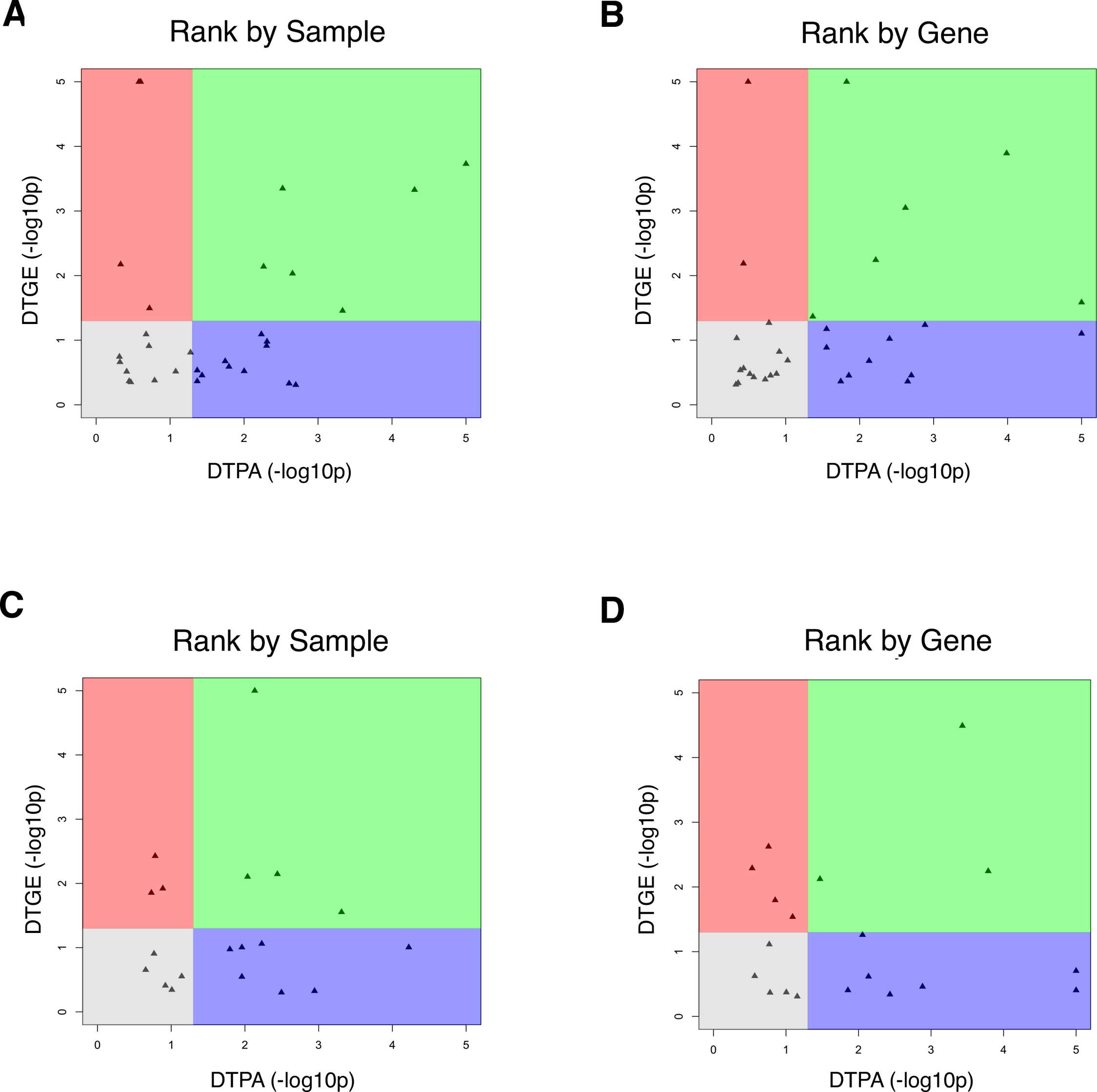

Supplement: S1 Fig — (A-D) Analysis for drugs targeting 75TRs in PC3 (A-B) or 19 TRs in HL60 (C-D) using either DTPA or DTGE for CMAP datasets. (A, C) For each TR with known inhibitors in the PC3 or HL60 datasets, we performed gene set enrichment analysis to test whether its DTPA or DTGE for its known inhibitors are significantly more inactivated or repressed compared to all other compound’s profiles and obtained p-values from each test. Then we plotted the distributions of the–log10 p-values for DTPA (x-axis) versus DTGE (y-axis). Each triangle represents a TR. A vertical and a horizontal line were drawn at p-value equals 0.05 for DTPA and DTGE, respectively, which divide the plot into four parts: green, blue, red, and grey. (B, D) For each TR with known inhibitors in the PC3 or HL60 datasets, we performed gene set enrichment analysis to test whether its DTPA or DTGE for its known inhibitors are significantly more inactivated or repressed compared to all other proteins and obtained p-values from each test. Then we plotted the distributions of the–log10 p-values for DTPA (x-axis) versus DTGE (y-axis). (TIF) [file pcbi.1005599.s005.tif]

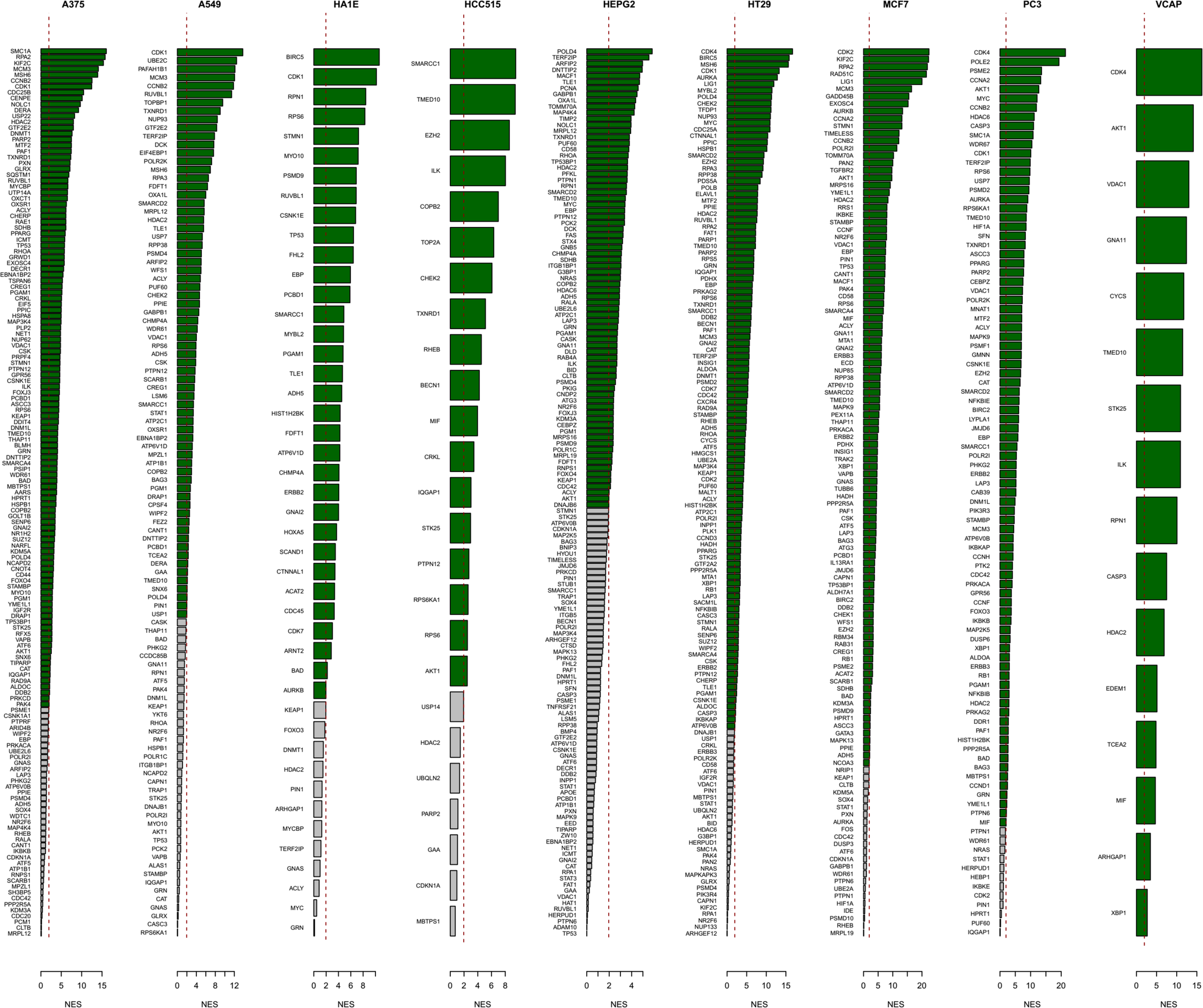

Supplement: S2 Fig — Results are shown cell line by cell line. Each bar is the analysis for one TR. A dotted line is drawn at NES = 1.96 (p = 0.05). TRs with significant enrichment (NES > 1.96; p < 0.05) are colored in green indicating the correlation between OncoLead CMoA inference and shRNA mediated TR silencing. Grey color indicates no significant enrichment. (TIF) [file pcbi.1005599.s006.tif]

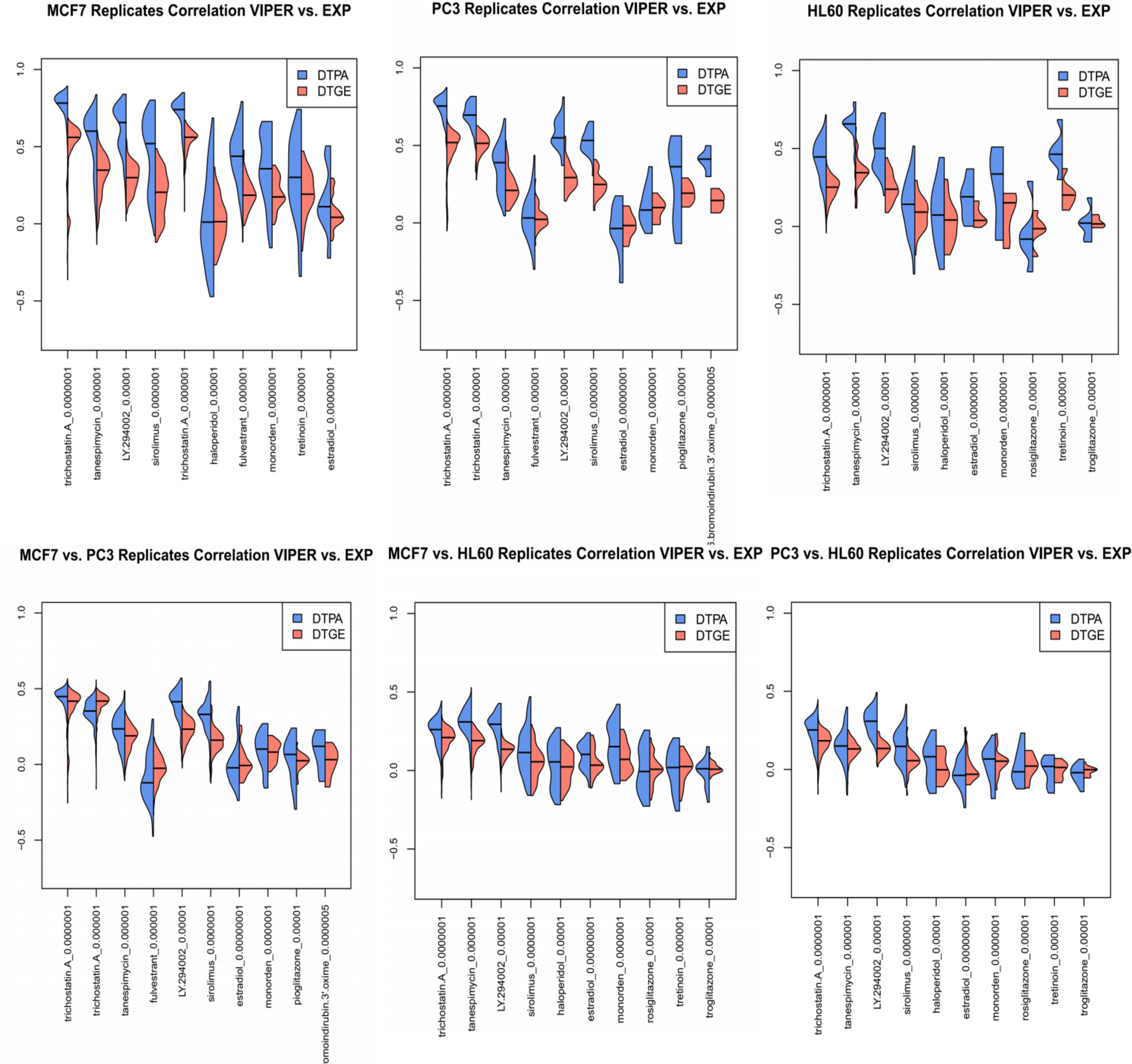

Supplement: S3 Fig — (TIF) [file pcbi.1005599.s007.tif]

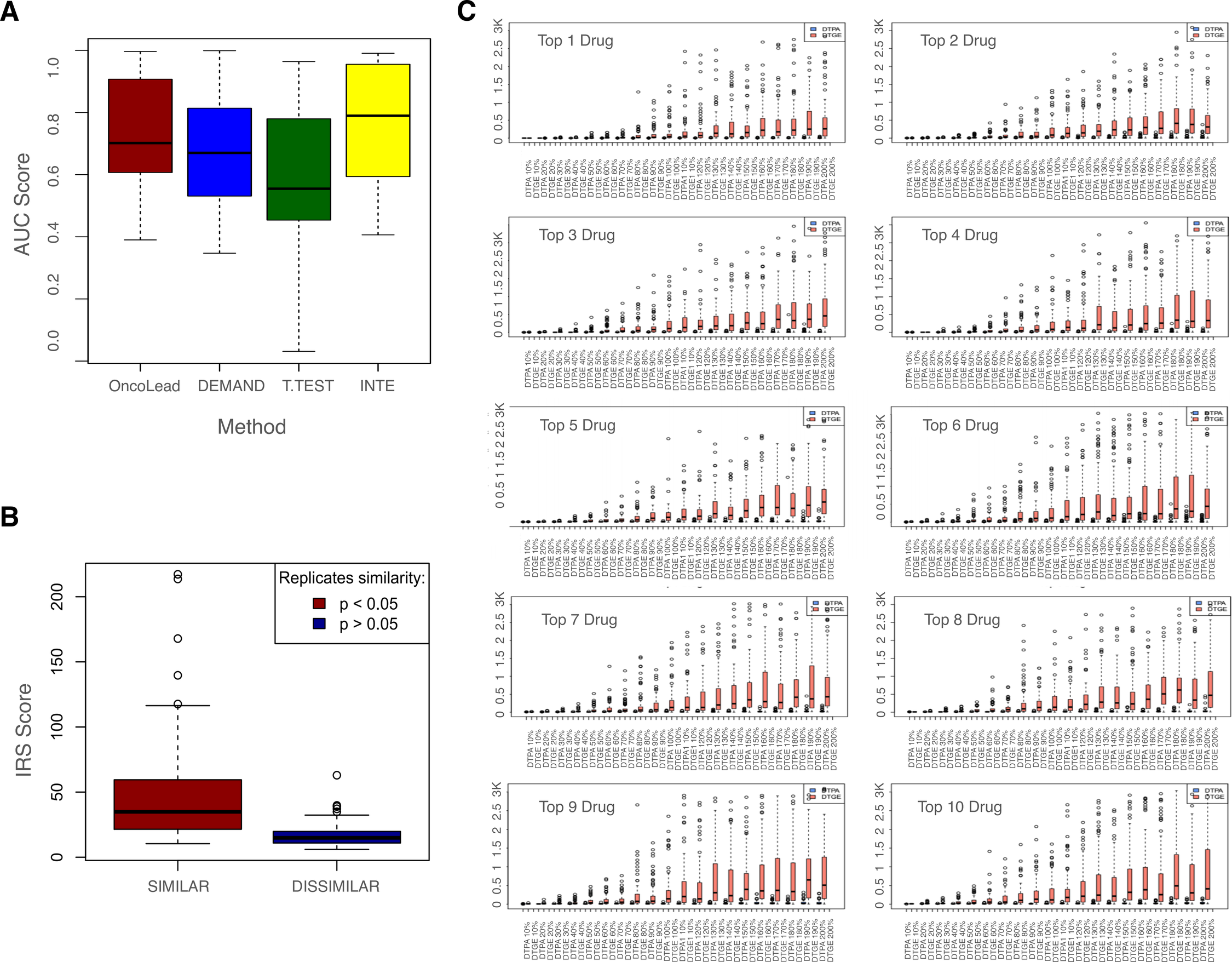

Supplement: S4 Fig — (A) Boxplot of the AUC score (area under the ROC curves as a function of the top predictions for identifying the known targets in the Dream dataset) using either OncoLead (red), DEMAND (blue), T-TEST (green) or integrating OncoLead and DEMAND result (yellow). (B) Boxplot of IRS scores for drugs whose replicates are significantly similar to each other (N = 76) and drugs whose replicates are dissimilar to each other (N = 94). (C) Box plot of the ranking positions of the top 10 drugs selected from CMAP-MCF7 data based on DTPA (blue) or DTGE (salmon) distances to a luminal breast cancer sample signature when adding Gaussian noise to the signature. For this analysis, we randomly select one luminal breast cancer gene expression profile from TCGA data set and add different levels of Gaussian noise to this profile. The Gaussian noise is a normal distribution centered in zero with the same length as the length of the gene expression profile. We generated 20 different levels of Gaussian noise, each has a different standard deviation (SD) ranging from 10% to 200% of the SD of the original gene expression profile. Then, for each different SD, we produce 1000 random gaussian noises and add each of them to the original gene expression profile and get 1000 gene expression profiles. Then for these 1000 modified gene expression profiles as well as the original profile, we did z-score transformation by minus the mean and divided by standard deviation of the TCGA basal breast cancer samples for each gene and obtained 1001 DTPA signatures. After that, we run OncoLead on each signature using breast cancer interactome to get DTPA for each signature. To find drugs that best reversing these signatures, we compute pearson correlation between CMAP-MCF7 drug induced DTPA and the 1001 DTPA and between CMAP-MCF7 drug induced DTGE and the 1001 DTGE. Ten drugs are selected which have the largest negative correlation to the original patient DTPA or DTGE signature. Finally, we plot the ranking posi [file pcbi.1005599.s008.tif]
